# Using a moot to develop students’ understanding of human cloning and statutory interpretation

**DOI:** 10.1177/0968533217726350

**Published:** 2017-09-12

**Authors:** Shaun D. Pattinson, Vanessa Kind

**Affiliations:** Durham University, UK

**Keywords:** Human cloning, statutory interpretation, mooting, student engagement, science education, somatic cell nuclear transfer

## Abstract

This article reports and analyses the method and findings from a 3-year interdisciplinary project investigating how the medium of law can support understanding of socio-scientific issues. Law represents one of the most important means by which society decides and communicates its values. Activities mirroring legal processes therefore have significant potential to inform, inspire and involve school students in exploring the conceptual, social and ethical issues relating to developments in biomedical science. This article focusses on an intervention-style study in which UK-based 16- to 17-year-old students role played a Supreme Court moot, developed by modifying a domestic appeal case concerned with whether the contemporary legislation covered the creation of cloned human embryos. We draw attention to how the science of cloning has been slightly misunderstood by the courts and in science materials provided to UK school students. We argue that moot-centred engagement activities offer great potential for science communication among post-16 students and, despite the limitations of the judicial process for addressing complex socio-scientific issues, such role plays aid development of scientific and sociolegal understanding, as well as enhancing students’ self-confidence and argumentation skills.

## Introduction

It is two decades since the birth of Dolly the sheep was announced in *Nature*.^1^ She was to become the most famous sheep in history, because she was a ‘clone’ in the sense of being (almost) genetically identical to another sheep. At the time, the President of the United States was quick to denounce human cloning as ‘morally unacceptable’^2^ and the United Kingdom’s Human Fertilisation and Embryology Authority (HFEA) soon declared that the technique required a licence under UK legislation and no treatment licence would be issued.^3^

In 2000, Sir Liam Donaldson, then the United Kingdom’s Chief Medical Officer, issued a report on stem cell research in which it was simply assumed that cloning fell within the Human Fertilisation and Embryology Act 1990 (hereafter, the 1990 Act).^4^ The Government’s response, proceeding on the basis that a cloned human embryo would fall within the Act, resulted in a judicial review action brought by Bruno Quintavalle on behalf of the Pro-Life Alliance: *R (Bruno Quintavalle) v. Secretary of State for Health* (hereafter, *Quintavalle*).^5^ The Pro-Life Alliance wished to get the courts to declare that if the technique used to produce Dolly were applied to human cells, it would not fall within the 1990 Act. This pressure group was, as its name implies, opposed to cloning and destructive use of embryos but was hoping that if parliament were forced to act, then the result would be a blanket legislative prohibition. This case took only 3 years to work its way through the courts. It succeeded at first instance, but Crane J.’s decision was reversed by the Court of Appeal and the House of Lords affirmed that reversal.

In theory, a cloned embryo could be created for the purpose of creating a human child (reproductive cloning) or for a purpose involving its destructive use, such as for research or to develop treatments (non-reproductive cloning). The former is more controversial but both are widely condemned by international instruments.^6^ By way of example, Article 18(1) of the European Convention on Human Rights and Biomedicine prohibits the creation of human embryos for research purposes (thereby prohibiting non-reproductive cloning and the research necessary for reproductive cloning) and Article 1 of its Additional Protocol on the Prohibition of Cloning Human Beings prohibits ‘[a]ny intervention seeking to create a human being genetically identical to another human being, whether living or dead’. The Protocol’s prohibition captures all cloning techniques, including the one used to produce Dolly, because ‘genetically identical’ is defined in Article 1(2) as ‘sharing with another the same nuclear gene set’. A study of 30 countries conducted in 2004 reported that none had permitted reproductive cloning, but non-reproductive cloning was either permitted or not clearly illegal in 13 of those countries.^7^

Neither the science nor the law has remained static. More than 20 different mammalian species have now been successfully cloned.^8^ Embryonic stem cells were derived from human embryos only a year after Dolly was announced^9^ and have now been derived from cloned human embryos.^10^ This is significant because stem cells could one day be used to treat a wide range of diseases for which there is currently no cure, such as diabetes, Parkinson’s disease and macular degeneration.^11^ Stem cells from cloned embryos have particular potential because embryonic stem cells are capable of becoming any of the 200 or so cell types in the human body and derivation from cloned embryos could enable them to be transplanted into a patient without triggering a negative immune response. Legally, non-reproductive cloning (including the creation of cloned embryos for stem cell research) remains legal in the United Kingdom and was licensed even before enactment of the Human Fertilisation and Embryology Act 2008.^12^ In contrast, reproductive cloning was subject to explicit legislative prohibition in the Human Reproductive Cloning Act 2001 and is now prohibited by provisions inserted into the 1990 Act (sections 3(2) and 3ZA(4)) by the 2008 Act.

A related technology, mitochondrial replacement therapy, aims to prevent the transmission of mitochondrial disorders from mother to child by replacing the mitochondrial DNA in the mother’s egg, or an embryo created from it, with that from a donated egg or embryo.^13^ This emerging therapy does not involve cloning, because any resulting child will not share nuclear DNA with any other embryo or existing person, but the relevant techniques use, or build on, the science of nuclear transfer.^14^ Thus, cloning technology is still developing. In 2015, the United Kingdom became the first country in the world to enact legislation permitting mitochondrial replacement therapy.^15^ In June 2017, the HFEA announced that it had approved its first application for use of this therapy to treat patients.^16^ It seems to us likely that the almost universal regulatory condemnation of reproductive cloning will face considerable political challenge if nuclear transfer technology develops to the point whereby the evidence suggests that it is as safe as more established forms of assisted reproduction, such as in vitro fertilisation (IVF).

This article will first provide an overview of the novel 3-year interdisciplinary project from which the empirical research in this article derives, with particular focus on the Supreme Court moot based on the *Quintavalle* case (hereafter, the Supreme Court moot). It will then revisit the science of cloning and show how it was slightly misunderstood by the courts in *Quintavalle* and in science materials provided to UK school students. The rest of this article is devoted to analysing data from the Supreme Court moot and its associated preparatory activities. First, arguments made by ‘Counsel’ and ‘Justices’ as played by the students are analysed and shown to display significant understanding of the science, innovative legal reasoning and some understanding of the underlying ethical issues. Secondly, data from questionnaires and interviews illustrate that although students learned appropriate science from participation in the project, in the case of one issue, this was short-lived. Also, data reveal significant positive impact on students’ self-confidence and argumentation skills emerged from preparation and presenting cases requiring them to speak in public.

## Overview of project activities

This article stems from work undertaken as part of a 3-year project, entitled ‘Human Cloning and Stem Cell Research through the Medium of Law’, funded by a Wellcome Trust People Award. Phase one activities (2013–2014) culminated in a ‘law-in-action workshop’ with post-16 students in which three fictional political parties engaged in two parliamentary debates on specific sections of a specially drafted Stem Cell Bill. Phase two activities (2014–2015) centred on a law-in-action workshop involving a moot on human cloning followed by a mock parliamentary debate on a proposed statutory instrument designed to permit mitochondrial replacement therapy.

This article is concerned with the Supreme Court moot on human cloning from phase two of the project.^17^ Six videos from 2 days of activities have been posted online.^18^ Day 1 included an ‘ethics activity’, which utilized a novel formulation of the ‘trolley problem’ thought experiment^19^; a ‘science activity’ in which students used Play-Doh to model relevant biological processes and scientific techniques; a lecture from an eminent researcher in relevant basic, translational and clinical science^20^; and various ‘law activities’ in which the relevant features of UK legal processes were explained. Following these, students were allocated into teams and asked to appoint members to specified roles. Access to an online learning environment enabled team members to liaise when preparing for the second day, which took place 2 weeks later. Day 2 included a Supreme Court moot on human cloning about which this article is principally concerned. Students’ understanding about human cloning and mitochondrial donation was probed prior to the moot (‘pre-moot’), immediately after the moot (‘post-moot’) and about 6 months after participation (‘delayed-post moot’).

For the Supreme Court moot, students were divided equally into two teams, namely, the ‘Pro-Life Team’ and ‘Secretary of State Team’. Each team appointed three researchers, responsible for preparing skeletal arguments and liaising with team members on the virtual learning environment between the 2 days; two counsel, senior and junior, to present the case in court; and three officials, who left their teams before preparation of the case and were trained to play five judges and the court usher.

Information provided to students included guidance notes on interpreting and using the law, and on role-playing researchers, counsel and court officials. Students were asked not to use legal materials beyond those provided, which included the ‘Reproduction and Embryology Act 1990’, which repackaged relevant sections from the 1990 Act into a form suitable for the moot, and heavily edited versions of Crane J.’s judgment and the judgment of the Court of Appeal in *Quintavalle.* None of the participants had studied law and students were not told that the moot was based on an actual case.

Section 1(1) of the Reproductive and Embryology Act 1990 mirrored that provision in the 1990 Act:

In this Act,

- embryo means a live human embryo where fertilisation is complete, and
- references to an embryo include an egg in the process of fertilisation, and, for this purpose, fertilisation is not complete until the appearance of a two cell zygote.

Section 2 set up and empowered a ‘Licensing Authority’, thereby capturing aspects of sections 5–10 of the 1990 Act. Section 3 provided for various offences, capturing the essence of section 3 of the 1990 Act. This section made it an offence to create a human embryo outside of the body or use it for any purpose, unless a licence has been granted or keep or use an embryo after the appearance of the primitive steak or after 14 days. A key provision, section 3(2)(a), mirrored section 3(3)(d) of the 1990 Act in its prohibition of ‘replacing a nucleus of a cell of an embryo with a nucleus taken from a cell of any person, embryo or subsequent development of an embryo’. Section 4 laid down conditions for licences, thereby covering aspects of Schedule 2 (activities for which licences may be granted) and Schedule 3 (consents for use of gametes or embryos) to the 1990 Act. Section 5 provided for the power to make regulations specifying the purposes for which a licence may be granted for research using embryos. This section was included so that the mock legislation had practical applicability without need for the more detailed provisions of Schedule 2 to the 1990 Act.

The Pro-Life team were instructed to argue that (1) the Dolly technique falls outside the Act and (2), if it does not, then section 3(2)(a) prohibits the technique. The first line of argument was to be presented by senior counsel. This turned on the definition of an embryo in section 1(1) and had been accepted by Crane J., whose judgment they were seeking to reinstate. The second line of argument, focussed on the prohibition of replacing the nucleus of an embryo with another nucleus in section 3(2)(a), was to be presented by junior counsel. This second argument was not mentioned in the edited judgments of the lower courts, save for their conclusion that it was to be rejected. The judgments were edited in this way to avoid unduly loading the debate in favour of the Secretary of State, who succeeded on this second point before Crane J. and the Court of Appeal.

The Secretary of State team were to argue that (1) the Dolly technique falls within the Act and (2) section 3(2)(b) does not apply to the technique. Again, the first argument was to be presented by senior counsel and the second by junior counsel. Their aim was to persuade the Supreme Court to uphold the decision of the Court of Appeal.

## Creating Dolly: The science of somatic cell nuclear transfer

Fertilization involves a sperm joining with an egg and developing into an embryo. Genetically, identical copies occur if that embryo splits before the development of the so-called primitive streak. That is to say that embryos produced by embryo splitting will be clones; they will be identical (monozygotic) twins. Dolly was created in a different way. She was the product of part of an egg joining with a body (somatic) cell. The nucleus was first removed from an egg. The nucleus-free (enucleated) egg was fused with a somatic cell taken from the mammary gland of a sheep by electric stimulation (it was the use of a mammary gland cell that gave Dolly her name; she was named after Dolly Parton). Chemical signals were then used to trigger the onset of embryonic development.

We have described the method used to create Dolly the sheep (hereafter ‘the Dolly technique’) as involving the transfer of a *somatic cell* into an enucleated egg. The standard nomenclature is ‘somatic cell nuclear transfer’, which suggests that the technique involves the transfer of the isolated *nucleus* from a somatic cell into the enucleated egg. The Donaldson report refers to the technique as one ‘in which the *nucleus* of an adult cell is fused with an egg which has had its nucleus removed’.^21^ The report goes on to provide a picture showing a ‘nucleus *taken from* adult (somatic) cell’ being inserted into an enucleated egg.^22^ This understanding was expressed by Crane J. (and Lord Phillips on appeal) in *Quintavalle*, who refers to ‘cell nuclear replacement’ as a process by which the ‘nucleus…*from* one cell is transplanted’ into an enucleated egg.^23^ The Dolly technique is similarly described by many medical law textbooks^24^ and, crucially for our engagement project, by learning materials provided to those studying biology within the General Certificate of Secondary Education (GCSE, taken at age 16), at Advanced Supplementary (AS) Level (taken at age 17) and Advanced 2 (A2) Level (taken at age 18). One GCSE textbook describes the method by which Dolly was created thus: ‘[t]he nucleus was taken out of a body cell from a different sheep’ and ‘[t]he body cell nucleus was put into the empty egg cell’.^25^ An AS Level textbook shows the ‘isolated nucleus’ being placed into an ‘enucleated egg cell’.^26^ GCSE and A2 Level examination specifications either do not provide details of the technique or do so inconsistently. For example, the Welsh Joint Examinations Council specification refers to ‘nuclear transplants from somatic cells into egg cells’,^27^ while EdExcel describes ‘removal of diploid nucleus from a body cell’^28^ and the Assessment and Qualifications Alliance states ‘[t]he nucleus from an adult body cell, e.g. a skin cell, is then inserted into the egg cell’.^29^

Professor Mary Herbert, an expert with direct laboratory experience of the process, delivered a lecture on day 1 of the workshop. She explained the technique as involving the fusion of an entire somatic cell with the enucleated egg.^30^ The scientific paper in which Dolly’s successful birth was first announced refers to the technique as ‘nuclear transfer’ throughout, and the abstract describes it as the ‘[t]ransfer of a single nucleus at a specific stage of development, to an enucleated unfertilized egg’.^31^ The ‘methods’ section states that the procedure involves the ‘[f]usion of the *donor cell* to the enucleated oocyte’.^32^ A later editorial in the same journal similarly states that ‘[t]o create Dolly, a mature cell from the mammary gland of one sheep was fused with the oocyte (egg cell) from another, from which oocyte the nucleus had previously been removed’.^33^ This is supported by other sources. Protocols from current cloning research make no mention of enucleating the somatic cell as part of the process,^34^ and the images from recently published papers graphically show that the nucleus is not removed from the somatic cell before being transferred.^35^

This insight into the science raises interesting questions. First, it suggests that the resulting cloned embryo will have mitochondria from *both* the egg provider and the somatic cell provider, which has been borne out in practice.^36^ Mitochondrial defects from either source could thereby be passed on, including those from the donor of the somatic cell, even if that person is male and would thereby not be able to pass on mitochondria by standard fertilization. Secondly, questions arise from the contrast between what is stated in student science materials (which also reflect what was understood by the court in *Quintavalle*) and the actual process for creating cloned human embryos in practice. We wonder if any student in the GCSE, AS or A2 Level assessment process has been penalized for suggesting that the somatic cell is fused with the enucleated egg. Thirdly, it raises an additional point relevant to the *Quintavalle* litigation, considered below in light of the fact that it was spotted by some students in the Supreme Court moot. Fourthly, this difference in method description provides a data point by which to measure the impact of this invention on the students’ understanding.

## Arguments of counsel and the court in the Supreme Court moot

Teams were asked to supply a ‘skeletal argument’ (written submissions) for distribution to opposing counsel and the Supreme Court Justices prior to the hearing. In the hearing,^37^ counsel for Pro-Life were invited to present oral arguments on the two issues, counsel for the Secretary of State were invited to present the counterargument and counsel for Pro-Life were then invited to respond. The Supreme Court Justices chose to question counsel only once the four main speeches had been presented; they retired to discuss the case without follow-up questions in response to Pro-Life’s closing submissions. The President of the Supreme Court gave the judgment of the Court with brief reasons: the panel decided 4:1 in favour of the Secretary of State on the first issue and unanimously in favour of the Secretary of State on the second issue. The dissenting judge on the first point provided reasons of her own and another Supreme Court Justice gave a brief concurring judgment on the second point. We will analyse the arguments on the two legal points separately.

### Issue 1: Whether an entity created by the Dolly technique falls within section 1(1)

The Pro-Life team argued that the specific wording of section 1(1), quoted above, did not apply to an entity created by the Dolly technique. After explaining that ‘[f]ertilisation involves an egg and a sperm fusing together to give a zygote’, the skeletal argument quoted the relevant provision and submitted that the Act did not anticipate the Dolly technique and section 1(1) should be interpreted ‘literally’, because there was ‘no intention to include it, therefore it doesn’t fall within the act’ and a purposive interpretation would be ‘an illegitimate extension of the act’. The skeletal argument also distinguished embryo splitting as a ‘natural form of cloning’ from the Dolly technique and explained that the latter ‘uses an egg where the nuclear DNA is removed and somatic cell (body) is placed inside where an electrostatic shock occurs to start development and therefore creating a cloned embryo’.

The oral presentation followed this structure but added moral rhetoric by opening with the submission that the technique invited ‘reflection to Frankenstein’s monster’ and closing with an appeal to the judges to ‘vote against the Dolly technique, not only for your moral conscience but also for your social duty’. The key argument was that:

The Dolly technique does not have two gametes, hence a two celled zygote is not made present. And with the knowledge of the Act saying fertilisation is not complete until the appearance of a two celled zygote, fertilisation does not occur. Therefore, it does not fall within the Act and is illegal. Also this technique is radically different from natural cloning. Indeed, it is even questionable if the same word ‘cloning’ should be used to identify the Dolly technique.

Both written and oral submissions display profound understanding of the relevant science. The explanation of the process in the written submissions follows that provided in the preparatory exercises, rather than by GCSE, AS Level and A2 Level materials (see above), because it refers to fusion of the enucleated egg with the ‘somatic cell’, rather than merely its nucleus. This science is then tied to the wording of section 1(1) and its references to ‘fertilisation’ and ‘zygote’.

The moral language used by senior counsel is consistent with the values attributed to their ‘client’. The team’s understanding of the consequence of a literal interpretation of section 1(1) was less clearly displayed, because while the written and oral submissions stated that their submitted view was that the Dolly technique falls outside of the Act, they suggest that use of the technique on human cells would therefore be ‘illegal’. This conclusion does not follow from their argument. Indeed, it would require rejection of their primary argument in favour of their alternative argument on section 3(2)(b).

The Secretary of State argued that the Dolly technique had not been specifically envisaged by Parliament but was nonetheless captured by a purposive interpretation of section 1(1). The skeletal argument presented bullet points without details, conceding that there are ‘[d]ifferences between artificial and natural fertilization of an embryo’ and the ‘act predates dolly technique by 7 years’ but submitting that the ‘act was created in order to pre-empt the formulation of these techniques’. Thus, while the ‘traditional definition of fertilisation is not applicable to the Dolly technique’, the law sought to regulate, rather than prohibit, such future developments.

The oral submissions eloquently advanced this line of reasoning. The Act, it was submitted, ‘was created after the 1984 Warnock report with the express will…to legislate for the potential creation of new ways for forming an embryo’. Reviewing the Act’s reliance on the word ‘fertilisation’ counsel submitted:

Fertilisation in its original and traditional route is taken to mean the fusing of two gametes – that would be the male sperm and female egg, both containing 23 chromosomes – to create a zygote of 23 pairs of chromosomes. Obviously, the Dolly technique differs slightly from this, in that the somatic cell already contains all 23 pairs.

But, counsel submitted, the ‘most essential element’ of the definition of an embryo in section 1(1) was that

the embryo itself is no different to an embryo created in the traditional sense; it still performs and divides in the same way and so it cannot be said to be said to be any different because it is qualitatively indistinguishable.

A purposive interpretation was to be adopted because

we must be pragmatic in realising that fertilisation is a term that moves rapidity with the progression of science, which itself is something which is extremely hard even to keep up with in our vernacular, let alone in legislation. So while it may feel in a certain sense that fertilisation is referring to the traditional sense of the word, we must defer and build upon [the] judgment of Lord Phillips, who said that we must add words to the legislation to say…fertilisation or any other technique…This counsel submits that that is not required, we must merely read into the [legislation] that it is the creation of an embryo of qualitatively indistinguishable characteristics from any other embryo created, rather than purely the meeting of gametes.

Like the Pro-Life team, the Secretary of State team displayed profound understanding of the science, specifically in referring to the chromosomal attributes of the human cells used in standard fertilization and cloning. In terms of legal understanding, their submissions not only identified the purpose of the Act but explicitly sought a purposive approach that differs from that adopted by Lord Phillips in the Court of Appeal.

The approach advanced by the Secretary of State team aligns with the published views of one of the authors of this article,^38^ but the students offered a differing explanation as to why it is better to give effect to the Act’s underlying purpose by construing the word ‘fertilisation’ purposively, rather than by reading words into the Act. This team submitted that ‘fertilisation is a term that moves rapidly’, thereby implying that scientists would now use the term to capture the process of creating a functional embryo by nuclear transfer. In contrast, the literature makes no such claims about non-legal usage of this word. Lord Phillips’ judgment refers to three gaps created by the Court of Appeal’s approach of reading the words ‘if it is produced by fertilisation’ into the Act’s definition of an embryo, which he dismissed on the basis that they lacked practical significance or could be cured by the Licensing Authority imposing equivalent requirements.^39^ The provisions of the ‘Reproductive and Embryology Act’ retained these gaps and included a section expressly permitting the Authority to impose any other conditions it considers appropriate. The first of the two most significant gaps can be found in section 3(2)(a) of the fictional Act, which captures the key attributes of sections 3(3)(a) and 3(4) of the 1990 Act by stating that a licence cannot authorise:

keeping or using an embryo after the appearance of the primitive streak, which it taken to have appeared in an embryo not later than 14 days from when the gametes are mixed.

If gametes are defined as sperm and eggs, then this provision does not apply to the Dolly technique, so such embryos are not subject to statutory restriction on the time during which they may be kept and used. The second significant gap can be found in sections 4(1) and (2) of the fictional Act, which captures the requirement that written consent be obtained from those providing ‘gametes’ for the creation of an embryo, which appears in Schedule 3 to the 1990 Act. If gametes are defined as sperm and eggs, then this is another provision that does not apply, as consent would not be required from the person who provides the somatic cell and is thereby cloned.

All three gaps identified by Lord Phillips could be avoided by taking the purposive approach to its logical conclusion and construing the terms ‘fertilisation’ and ‘gametes’ to give effect to the Act’s underlying position on the status of the embryo.^40^ The Warnock Report upon which the 1990 Act was based explicitly took the view that ‘the embryo of the human species ought to have a special status’, albeit not the ‘same status’ as a living child or an adult,^41^ and the long title of the 1990 Act (which was reproduced in the mock legislation used for the moot) states that its purpose is to ‘make provision in connection with human embryos and any subsequent development of such embryos’. Invoking the purpose of regulating use of embryos in recognition of their special status would require ‘fertilisation’ to be interpreted as the creation of a functional embryo by the joining of genetic material and a ‘gamete’ to be defined accordingly. Such an approach would also have provided some future-proofing to the legislation by capturing the creation of functional gametes from stem cells (‘*in vitro* derived’ or ‘artificial’ gametes).^42^ The Secretary of State team moved towards such an approach, which supports our view that it would have been an appropriate way for the appeal court to give effect to the identified purpose of the 1990 Act in light of the science.

The majority of the student Supreme Court Justices upheld the Court of Appeal’s conclusion on the first issue, but the President’s reasons did not identify the mechanism by which the purposive interpretation was to be given effect. It was simply asserted that ‘[t]he definition should be changed because an embryo created by [the] Dolly [technique or by fertilisation] is the same’. The definition of an embryo in the Act ‘needs to keep up with scientific developments as stated before’, which seems to be a reference to the argument advanced on behalf of the Secretary of State team. The President thereby used language more appropriate for a legislature than an appeal court. In contrast, the dissenting Justice opined that ‘Parliament did not foresee such a technique…and therefore to say that the Act covers this is not a correct view of the law’. This corresponds to the view of the majority of academic commentators on *Quintavalle.* ^43^

In the actual decision, the House of Lords adopted an alternative approach to construing section 1(1)(a) of the 1990 Act. According to Lord Bingham, the words referring to fertilization ‘were not intended to form an integral part of the definition of embryo but were directed to the time at which it should be treated as such’.^44^ In other words, the provision was to be read as specifying no more than when a fertilized egg was to be regarded as an embryo. This left the Licensing Authority (the HFEA) to plug the gaps identified by Lord Phillips with its licensing terms, rather than closing those gaps by applying the Act’s existing provisions to them.

### Issue 2: Whether section 3(2)(a) prohibits the application of the Dolly technique to human cells

The second issue turned on the meaning of the section 3(2)(a) prohibition on ‘replacing a nucleus of a cell of an embryo with a nucleus taken from a cell of any person, embryo or subsequent development of an embryo’.

The Pro-Life team’s argument was that the purpose of this provision was to prohibit nuclear transfer. The skeletal argument referred to ‘276 aborted foetus[es] used to create Dolly’ and the ‘need [for] more extensive research to see the effect on humans’. The oral submission elaborated thus:

…Pro-Life aims to keep morally clean societies. This procedure goes against section 3(2)(b) of the Act due to its removal of nuclear DNA. And it is common belief that it is not at this stage that the nuclear DNA is removed, which falls into question, but the fact that the result is the same. The legislation was put into place to ensure the safety of human life and subsequent development of an embryo and therefore is prohibited. The legislation itself was created before the procedure came about and so therefore there is no way that it would be applied to it. And here is no way that it could fall within the Act, therefore the procedure is wrong and ethically unsafe.

These submissions display scientific and legal misunderstandings. Dolly was the only successful birth from 277 attempts to transfer somatic nuclear DNA into an egg and the only one that developed into a fetus.^45^ The effect of the technique falling within section 3(2)(b) would be that it is prohibited, rather than the Act not applying to it. Nonetheless, a more charitable reading was that the Pro-Life team were seeking to argue that the purpose of the provision was to prohibit cloning by nuclear transfer on the basis that it is ‘wrong and ethically unsafe’, which would apply with equal force to cloning by the specific technique used to produce Dolly.

The Secretary of State team argued that section 3(2)(b) did not apply. The written submission declared that the ‘Dolly technique does not involve an unfertilized egg, it uses an embryo’. Junior counsel submitted that the provision should be interpreted literally because, in contrast to section 1(1), it is ‘highly specific and its purpose is to…[regulate] direct nuclear transfer techniques’. The Dolly technique ‘involves removing the nucleus from an unfertilised egg and the insertion or fusion of a somatic cell with the electric shock’. The language of section 3(2)(b) was then analysed. It was submitted that the Dolly technique ‘involves an unfertilised egg not an embryo’ and

The section states that the nucleus is removed and replaced by another nucleus. However, once again this does not apply to the Dolly technique, as the nucleus is removed then a fertilised egg is fused with a *complete* somatic cell, not simply a nucleus alone. (Student speaker’s emphasis)

Further, the Pro-Life team’s ‘opposition to this cloning is purely based upon the abuse of the technique’ and this overlooks potential positive uses for cloning, such as creating ‘organs that are specifically tailored for each person and prevent organ rejection and the need for expense immunosuppressant drugs’. These submissions display profound understanding of the science and went beyond the analysis actually adopted by the House of Lords. The revised understanding of the science gave the Secretary of State team the opportunity of arguing that the Dolly technique fell outside of the provision because not only did it not involve ‘replacing a nucleus of a cell of an embryo’, but the material transferred into that embryo was not strictly ‘a nucleus taken from a cell of any person, embryo or subsequent development of an embryo’. This supports our view that a moot can be an effective way of directing student’s minds to the details of biomedical science and shows the importance of the facts as understood by the court.^46^

The students’ Supreme Court unanimously agreed with the Secretary of State team’s submissions. The President stated that this was ‘because there was not an altering of the embryo, as they were altering an unfertilised egg. They were not swapping the nucleus of two, they were inserting another cell’. This displays an understanding of the Dolly technique as presented by both counsel, rather than following the wording of school science materials and the edited judgments of the lower courts. No attempt was made to contrast and explain the literal approach to section 3(2)(b) in light of the purposive approach taken towards section 1(1). Another Justice added a concurring speech stating that the Dolly technique ‘did not break any rules set by the Act’, which shows that ‘Parliament did mean to allow this technique in the future’. This refers to Parliament’s intention but does not really add further explanation. As an exercise in science understanding, however, the moot was successful.

Lord Bingham, in the actual decision of the House of Lords, held that the Dolly technique ‘does not involve “replacing a nucleus of a cell of an embryo” because there is no embryo until the nucleus of the recipient cell is replaced by the nucleus of the donor cell’.^47^ Thus, the House of Lords ruled, section 3(3)(d) of the 1990 Act did not prohibit the application of that technique to human cells. An alternative approach would have been to accept that the provision was to be interpreted purposively, rather than literally, but argue that the purpose was, at least in part, to give effect to the Act’s underlying position on the status of the embryo.^48^ Replacing the nucleus of an embryo involves the destruction of an embryo, whereas creating an embryo using the Dolly technique does not. The purpose of either prohibiting cloning or prohibiting nuclear replacement as such also encounters a boot strap problem: we are required to read words into a section to fulfil the purpose of that section when that purpose itself is said to derive from that very section.

## Analysis of empirical data obtained from the student participants

### The student sample

The sample comprised 51 16- to 17-year-old (Year 12, ‘Lower Sixth’) students drawn from four state-funded comprehensive schools in North East England. All are 11–19 schools (including one for children and students aged 4–19) serving areas that include communities with participation rates in post-16 and post-18 education that are lower than the national average. Thus, some participants were apprehensive about visiting the university that provided the project locus and working with students from other schools. Table 1 shows background information about the students’ GCSE results, broad post-16 AS subject choices and reasons for participating in the project. Thirty students (about 59%) were female and the remainder male. Table 1 shows that about 70% of participants had strong GCSE backgrounds, indicated by the proportions with A*/A and B/A grades. Anecdotal evidence from school staff indicated the participants represented their ‘most able’ Year 12s. Nearly 70% of students were studying one or more AS science subjects: these subdivide further into 38% of the cohort who were pursuing sciences alone, 21% studying science and ethics and 10% a mix of science and humanities subjects. In promoting the project to schools, we emphasized that the project was suitable for participants studying all subjects. However, within schools, the project was strongly perceived as more relevant to science- than humanities-oriented students. This is consistent with a relatively high proportion (about 45%) of students taking part to enhance their science knowledge. Nevertheless, Table 1 shows that 13 students (about 26%) participated to strengthen their knowledge of ethics or debating.

**Table 1.** Student participants’ backgrounds: GCSE grades AS subject choices and reasons for participating.

GCSE grades (%)		AS subjects (%)		Reason for participating (%)	
Mostly A*/A	31.4	Sciences	66.6	Science knowledge	45.1
Mostly B/A	39.2	Humanities	31.4	Ethics knowledge	15.7
Mostly B/C	21.6	No response	2.0	UCAS application	13.8
No response	7.8			Debating knowledge	9.8
				Other	7.8
				No response	7.8
Total	100.0		100.0		100.0

GCSE: General Certificate of Secondary Education; AS: Advanced Supplementary; UCAS: Universities and Colleges Admissions Service.

*Note*: *N* = 51.

## Data collection and analysis

Data relating to students’ understanding of cloning were obtained via a questionnaire comprising six multiple choice items. Of these, two questions (see Appendix 1) were used to elicit evidence of students’ understanding of the Dolly technique and human clones. Students were invited to respond to these questions on three occasions: immediately prior to participating in day 1 (science activities) abbreviated to ‘pre-moot’; immediately post-participation in day 2 (‘post-moot’); and in January 2016, approximately 6 months post-days 1 and 2, referred to as ‘delayed-post moot’. Students gave consent in writing to collect and report data, with appropriate assurances given regarding data protection. Data were collected in accordance with the British Education Research Association (2011) code of practice.^49^ No questionnaire data were stored electronically in any format that permits identification of individuals and/or connects roles played directly with questionnaire responses. The study received ethical assent from appropriate committees within the authors’ university.

The ‘creating Dolly’ question (Appendix 1) offers four options. Students selected the statement they thought best describes the technique used to create Dolly. Statement B is correct, that is, best matches Wilmut et al.’s description of the technique.^50^ Statement D represents science presented in textbooks used in teaching GCSE and Advanced Supplementary/A2 Level Biology (described above). In reporting, selecting statement D is graded ‘partially correct’. Statements A and C were devised by the authors as distractors. Selection of A or C is graded ‘incorrect’. Students’ responses to creating Dolly at the three data collection points are shown in Table 2.

**Table 2.** Students’ responses to the creating Dolly question pre-, post- and delayed-post moot.

Choose one statement which best describes the method used to create Dolly the sheep:	Response type	Pre-moot (%)	Post-moot (%)	Delayed-post moot (%)
The nucleus was removed from an egg, which was then fused with a mammary gland cell by electrical stimulation	Correct	43.1	66.7	33.3
The nucleus was removed from an egg, which was then fused with the nucleus from a mammary gland cell by electrical stimulation	Partially correct	29.4	9.8	5.9
An egg was fused with a sperm by electrical stimulation	Incorrect	19.6	0.0	31.4
The nucleus was removed from an early embryo which was then fused with the nucleus from a mammary gland cell by electrical stimulation				
No response		7.9	23.5	29.4

*Note*: *N* = 51.

The ‘human clone’ question (Appendix 1) offers four options. Students selected any combination they believed to be correct. The only correct response is B. Selection of B with statement D or selection of statement D alone indicates misunderstanding of human clone DNA at the genetic level, as the respondent does not realize that mitochondrial DNA may differ. This is reported as ‘Genotype error’. Selection of statement B and/or D with either A or C indicates misunderstanding of human clones at the phenotypic level, as the respondent does not realize that cloned human beings would have different external characteristics. This is reported as ‘Phenotype error’. Selection of either A or C alone or both A and C implies no understanding of human clone DNA. This response type is reported as ‘incorrect’. In practice, all students selected only one or two statements when responding to the human clone question on any data collection occasion. Students’ responses to this question are shown in Table 3.

**Table 3.** Response codes used in analysis of students’ responses to the human clone question.

Lisa and Kylie are clones. Which of these statements are true about Lisa and Kylie? Please tick all you think are true.	Response code			
	Correct	Genetic error	Phenotype error	Incorrect
They will share the same nuclear DNA.	X	X	X	
All their DNA will be identical.		X	X	
In appearance, Lisa and Kylie will be impossible to tell apart.			X	
Their memories, fingerprints and personalities will be identical.			X	X

The software IBM SPSS (version 20) was utilized to facilitate data analysis. Each student was assigned a unique anonymous identifier. Individual responses were coded as described above and entered into a database for analysis. *χ* ^2^ values were calculated for a 3 × 3 table with 4 degrees of freedom (DOFs; creating Dolly question) and for a 3 × 4 table with 6-DOF (human clone question). The *χ* ^2^ test is used to establish whether factors other than chance alone may be responsible for the distribution of data across selected variables, in these examples, the range of students’ responses to each question pre-, post- and delayed-post moot as shown in Tables 2 and 4. Note that ‘no response’ was excluded from these calculations.

**Table 4.** Students’ responses to the human clone question.

Response	Pre-moot (%)	Post-moot (%)	Delayed-post moot (%)
Correct	23.6	43.1	43.1
Genetic error	47.1	15.7	17.7
Phenotype error	13.7	7.7	9.8
Incorrect	7.8	0.0	0.0
No response	7.8	23.5	29.4

*Note*: *N* = 51.

Delayed-post moot semi-structured interviews took place with small groups of students in their schools about 6 months later, when delayed-post moot questionnaire data were also collected. Students were asked to discuss their views about the events, including what they found ‘good’ of ‘lasting benefit’ as well as their ‘outstanding memories’ and what they had learned about debating, the science, ethics and any other aspects. The interviewer probed further according to points raised by specific student groups. Interview data were analysed by content analysis procedures.^51^ Students’ views are reported in two broad categories: content, structure and organization and personal impact on participants. Subthemes within content, structure and organization are the debates; legal, scientific and ethical knowledge; and integration of knowledge. Subthemes within personal impact are transferrable skills, personal confidence and wider contributions.

## Students’ understanding of the Dolly technique and human clones

Table 2 shows data relating to students’ responses to the multiple-choice question posed in the questionnaire (Appendix 1).These data show that about 40% of the students held correct ideas pre-moot, a figure that increased immediately post-moot to about two-thirds. Delayed post moot figures suggest this understanding was not retained 6 months later, with students reverting to an incorrect response, and fewer correct responses than the pre-moot level. We need to bear in mind that students may have guessed their answers at each stage and that by the delayed-post point may have been fatigued by the project, so did not read the question statements carefully. However, the *χ* ^2^ value of 31.63 is significant at the 0.01 level (*ρ* = 0.00001). This means that there is smaller chance than 1/100 that the responses are impacted by chance alone, that is, other factors are more likely than chance to be responsible for the observed response pattern. For example, we note that the correct definition provided in the question differs from the definition of the Dolly technique used in biology examination specifications,^52^ and this may have been presented to students studying science in the intervening period.

Students’ response patterns were investigated further. Positively, 10 students (about 20%) gave incorrect responses pre-workshop, but changed to a correct response post-workshop and retained this response delayed-post. Of these 10 students, 7 were studying science subjects. However, 16 students (about 31%) gave incorrect delayed-post responses. Of these, 11 had given incorrect responses pre-workshop and correct responses post-workshop. The 16 students included 9 studying science and a further 3 science and humanities subjects. Recall of the precise detail involved in the Dolly technique is not only challenging but may have been impacted negatively by the provision of incorrect definitions within AS Biology specifications.

Table 3 presents the response codes relating to students’ responses to the question on the nature of human clones (Appendix 1).

Students were required to respond by selecting all statements they believed to be true. In fact, only the first statement is correct. Students selecting the top two statements hold a ‘genetic’ error, failing to understand that mitochondrial DNA differs from individual to individual (outside the same maternal line). Students selecting any other combination make a phenotype error. Those selecting the last statement hold a misunderstanding about the meaning of ‘clone’ akin to that presented in some science fiction.

Table 4 presents students’ responses to the human clone question. These show that about one-quarter held correct views prior to the moot, a figure which increased to over 40% post-workshop. This level was retained delayed-post moot. Nearly half of respondents demonstrated genotype errors pre-moot, suggesting they had not understood the significance of mitochondrial DNA in determining individuality. This response type is consistent with GCSE science teaching regarding identical twins. In post- and delayed-post workshop, this figure dropped to about 16% or about eight students. Phenotype errors were less frequent pre-workshop but also decreased to five students in the delayed-post workshop data. These data suggest that human cloning, as probed by this question, was relatively easier to understand and recall 6 months post-workshop.

The *χ* ^2^ value for these data is 21.59, and *ρ* = 0.001439, significant at the 0.01 level (6-DOF). This suggests that factors other than chance are responsible for the observed response pattern. For example, we note attrition of about 30% of students responding to the delayed-post questionnaire, due to factors such as change of school, failure to complete their AS studies successfully and not reporting to take the questionnaire for the third time.

## Students’ delayed-post moot group interview responses

Delayed-post workshop interview data generate insights into the personal impact of participation on students as well as in-depth opinions about the content, structure and organization of the events.

### Content, structure and organization

The moot and parliamentary debates showed students how law is made and interpreted, which was regarded as ‘technical and complex’. The wide range of roles was also appreciated, as this ‘helped to get more people involved’. Students were surprised by the tight structure imposed on the debates, noting that this gave an ‘official’ tone that ensured all views were heard, for example, ‘I learned about the structure of official debates…it wasn’t just a bunch of people in a room, yelling at each other…it was much more official’.

Counter-intuitively, the debate structures generated a sense that students felt able to contribute free from concern about personal redress, for example, ‘with [the debate] not being personal, everyone felt they could say something…you could say what you wanted’. This contributed to students’ enhanced personal confidence, discussed below.

Some students reported that they found the formality of the debates in terms of the language and ‘actual etiquette’ challenging, but noted that this gave a sense of being ‘really professional’. One student said using this language made him feel ‘more elaborate’, while another appreciated this ensured ‘arguments could be put forward in a more mature way, rather than dissolving’.

An aim for the event was to exemplify law, science and ethics intersecting. That this was apparent to participants is seen in comments such as ‘these two worlds [science and law] co-existing then overlapping – I loved seeing it’ and ‘realising science is affected by religion and ethics…having to see all the things together and symbiotically so you get the whole picture’. Depending on their background, different aspects of the workshop impacted more significantly on some students than others. For example, two students not studying science said, ‘the ethics was more interesting’ and ‘the experience gave an opportunity to visit my ideas of how we think in a moral perspective’. Science-oriented students appreciated the detailed content of the academic lecture, which gave ‘more depth’ to their knowledge. In general, participants had heard of the Dolly technique but had not realized that this had been discussed in law. One student explicitly stated, ‘It’s given me a bigger insight into medical ethics which has been really helpful’.

### Personal impact on participants

Students stated that participation in the project contributed to their developing a range of transferrable skills. A constant theme was working in a team or group with students they had not previously met. For example, ‘[We] were put into groups with people we didn’t know and managed to create a full debate…’ and ‘we were submerged with people we don’t know and straightaway had to work with [them], so that was good’. Students commented that ‘normal’ school provided limited opportunities for participating in ‘academic team work’. They found working in teams, ‘compiling questions, answers and speeches’, ‘looking at both sides of an argument’ and ‘discussing intellectually’ highly beneficial. Other skills mentioned frequently included reading, analysing and interpreting advanced material and developing public speaking abilities that required explanations of complex information. One student stated that the project helped him ‘structure an argument…‘ and that when he subsequently attended a university entrance interview he could ‘respond and disagree, but remain courteous’.

Many participants stated that the project impacted positively on their personal confidence. For example, one student said the event helped him overcome fears about ‘speaking in front of people’; another said that she ‘prepared the speech and…gave the speech…I didn’t think I would be able to do it…it gave me confidence’. Another student stated, ‘I was jumping to a higher skill level than I thought I had and obviously in front of people [I didn’t know]’.

Participants also noted that confidence increased gradually as debates progressed. For example, ‘At the start everyone was a bit shy and wary…then everyone wanted the microphone. They wanted to talk and express opinions…it was getting intense’. More precisely, students found that actually having to argue made them think carefully and rapidly, for example, ‘you have to think critically and on the spot…’, while another realized, ‘I’m going to have to defend my case…you have to be attentive to what your colleagues and the opposition are saying’. The importance of understanding material thoroughly to enable a sound defence and argument to be made was also apparent, as one student noted, ‘I had to stand by my argument…it’s important to be consistent…you can’t argue for something fully unless you have a good understanding of it’.

Interviewees commented on the wider contributions that participation in the workshop had provided. Several students used scientific content on mitochondrial donation as the topic for their extended project qualifications.^53^ Another made direct contact with the academic lecturer post-workshop and arranged to visit her laboratory for a period of work experience. One student reported she gained the knowledge to cite mitochondrial donation as an example of ‘recent science’ on attending an information event about biomedical sciences at a Russell Group university. Her interest in the topic had also led her to attend a public lecture. The lecture that formed part of the science preparation day was reported as highly influential, supporting students in confirming their subject choices and showing ‘what the next level [university] would look like’.

## Conclusion

Close analysis of the submissions and judgments made in the Supreme Court moot, and the data from questionnaires and post-intervention interviews, provides evidence of the strength and limitations of the moot procedure for engaging students with the socio-ethical issues relating to developments in biomedical science. A moot can be an effective means of both science communication and facilitating understanding of different ethical views. Other teaching tools methods may, however, be better for advancing ethical understanding than a bipartite moot that provides little opportunity to role play or examine a wide plurality of ethical views.^54^ Moots are, by their nature, focussed on narrow legal/regulatory issues. What is surprising is that our cohort of 16–17 year old students, with very little training in legal method, were able to advance well-constructed legal arguments, including arguments not considered in the litigation on which the Supreme Court moot was based. One of the principal arguments advanced relied on the revised understanding of the Dolly technique taught to the students during the day 1 science activities.

Mooting as a student learning activity assisted the participant students’ self-confidence and argumentation skills. The data also demonstrate the potential of mooting as a tool for science education. The Supreme Court moot successfully increased student understanding of the nature of a clone by significantly reducing the numbers making genetic or phenotypic errors. This percentage of correct views was retained 6 months later (see Table 4, above). Student understanding of the actual process used to create Dolly was similarly improved by the Supreme Court moot, but, less positively, data suggest that that understanding was not retained 6 months later (see Table 2, above). We suggest this failure to retain understanding over time may, at least in part, be attributed to the technicality of this narrow issue and note that the day 1 science activity material contradicts the description provided in school learning materials, to which students may have been subsequently exposed. Further, we attempted no revision process prior to the delayed post-moot interviews. Also, students questioned might not have held a direct role in the Supreme Court moot: day 2 was devised so that those with direct roles in the afternoon parliamentary debate on mitochondrial replacement therapy did not also play direct roles in the Supreme Court moot.

Our research provides incidental data relevant to contemporary research on gender representation in the legal professional and judiciary.^55^ While over half (59%) of our cohort were female, the students appointed men as both sets of senior and junior counsel and men similarly dominated the party leadership roles in the associated parliamentary debate (i.e. party leaders, deputy party leaders and speaker makers). We observed the students’ appointment process, in which they chose to select the first of their cohort to volunteer. The resultant gender disparity occurred despite our expert scientific lecture being given by a woman and the project team coordinating the student activities being gender balanced. We had, however, initially underplayed the significant role played by the judges in the Supreme Court moot (and speaker in the parliamentary debate) and arranged for those roles to be appointed from the last set of appointments with less inspiring titles (‘court officers’ and ‘parliamentary officers’, respectively). Student selection of men to play what were apparently the leadership roles, resting as it did on the greater eagerness of men to volunteer for those roles, leads us to suspect that gender-role stereotypes may be established by the age of 16. If this is so, then efforts to address gender disparities need to start earlier than at university level.

Overall, we conclude that moots have significant potential as tools for education and engagement with biomedical science. We show that moots assist school students who have not formally studied law to assimilate complex scientific information and engage in subtle argument on challenging scientific and legal issues.

## Appendix 1

### Question probing understanding of the Dolly technique

**Choose one statement** which **best describes** the method used to create Dolly the sheep:
  □ An egg was fused with a sperm by electrical stimulation.
  □ The nucleus was removed from an egg, which was then fused with a mammary gland cell by electrical stimulation.
  □ The nucleus was removed from an early embryo, which was then fused with the nucleus from a mammary gland cell by electrical stimulation.
  □ The nucleus was removed from an egg, which was then fused with the nucleus from a mammary gland cell by electrical stimulation.

### Question probing students’ understanding of human clones

Lisa and Kylie are clones. Which statements are true about Lisa and Kylie? Please **tick all statements you think are true**.
  □ In appearance, Lisa and Kylie will be impossible to tell apart.
  □ They will share the same nuclear DNA.
  □ Their memories, fingerprints and personalities will be identical.
  □ All their DNA will be identical.

## Notes

1. I. Wilmut et al., ‘Viable Offspring Derived from Fetal and Adult Mammalian Cells’, *Nature* 385 (1997), p. 810.

2. President Bill Clinton quoted in J. Harris, ‘Germline Modification and the Burden of Human Existence’, *Cambridge Quarterly of Healthcare Ethics* 25(1) (2016), pp. 6–7.

3. Human Fertilisation and Embryology Authority and Human Genetics Advisory Commission, *Cloning Issues in Reproduction, Science and Medicine: A Consultation Document* (London: HMSO, 1998), para 2; HFEA and HGAC, *Cloning Issues in Reproduction, Science and Medicine* (London: HMSO, 1998), para 3.4.

4. Department of Health, *Stem Cell Research: Medical Progress with Responsibility. A Report from the Chief Medical Officer’s Expert Group Reviewing the Potential of Developments in Stem Cell Research and Cell Nuclear Replacement to Benefit Human Health* (London: Department of Health, 2000), paras 13–14.

5. [2003] UKHL 13 (hereafter, HL); affirming [2002] EWCA Civ 29 (hereafter, CA); reversing [2001] EWHC 918 (Admin) (hereafter, Admin).

6. See further S. D. Pattinson, ‘Reproductive Cloning: Can Cloning Harm the Clone?’, *Medical Law Review* 10(3) (2002), p. 295.

7. S. D. Pattinson and T. Caulfield, ‘Variations and Voids: The Regulation of Human Cloning Around the World’, *BMC Medical Ethics* 5 (2004), p. 9. Available at: www.biomedcentral.com/1472-6939/5/9 (accessed 7 August 2017).

8. J. B. Cibelli, ‘Human Somatic Cell Nuclear Transfer is Alive and Well’, *Cell Stem Cell* 6 (2014), p. 699.

9. J. A. Thomson et al., ‘Embryonic Stem Cell Lines Derived from Human Blastocysts’, *Science* 282 (1998), p. 1145.

10. M. Tachibana et al., ‘Human Embryonic Stem Cells Derived by Somatic Cell Nuclear Transfer’, *Cell* 153 (2013), p. 1228; Y. G. Chung et al., ‘Human Somatic Cell Nuclear Transfer Using Adult Cells’, *Cell Stem Cell* 14(6) (2014), p. 777.

11. See, for example, I. Sample, ‘Stem Cell Therapy Success in Treatment of Sight Loss from Macular Degeneration’, *The Guardian*, 15 October 2014.

12. See HFEA, ‘HFEA Grants the First Therapeutic Cloning Licence for Research’, 11 August 2004. Available at: www.hfea.gov.uk/758.html (accessed 24 February 2017).

13. See Nuffield Council on Bioethics, *Novel Techniques for the Prevention of Mitochondrial DNA Disorders: An Ethical Review* (London: Nuffield Council on Bioethics, 2012); HFEA, *Mitochondrial Replacement Consultation: Advice to Government* (London: HFEA, 2013).

14. ‘Maternal spindle transfer’ involves removing the nuclear DNA from a woman’s egg and placing it into an enucleated donor egg. ‘Pronuclear transfer’ involves placing the pronuclei from a fertilized egg into a donated fertilized egg from which the pro-nuclei have been removed.

15. The Human Fertilisation and Embryology (Mitochondrial Donation) Regulations 2015/572.

16. HFEA, ‘HFEA Statement on Mitochondrial Donation’, *HFEA Press Release*, 29 June 2017. Available at: www.hfea.gov.uk/about-us/news-and-press-releases/2017-news-and-press-releases/hfea-statement-on-mitochondrial-donation (accessed 7 August 2017).

17. Anyone interesting in running a refined version of the moot described in this article should consult: S. D. Pattinson et al., *Human Cloning Activity Pack* (Durham CELLS, 2017). Available at: www.dur.ac.uk/cells/packs (accessed 7 August 2017).

18. Available at: www.dur.ac.uk/cells/engagement/scr/stream2 (accessed 7 August 2017).

19. Seminally, P. Foot, ‘The Problem of Abortion and the Doctrine of Double Effect’, *Oxford Review* 5 (1967), p. 5.

20. Professor Mary Herbert. Available at: www.ncl.ac.uk/igm/staff/profile/mary.herbert (accessed 7 August 2017).

21. Department of Health, *Stem Cell Research*, para 1.2 (emphasis added).

22. ibid., para 2.26 (figure 2) (emphasis added).

23. Admin [15], CA, [4], [11] and [35].

24. S. D. Pattinson, *Medical Law and Ethics*. 4th ed. (London: Sweet and Maxwell, 2014), p. 346; J. Herring, *Medical Law and Ethics*. 6th ed. (Oxford: OUP, 2016), p. 415. The description has been modified in the latest edition of the former: S. D. Pattinson, *Medical Law and Ethics*. 5th ed. (London: Sweet and Maxwell, 2017), p. 350.

25. D. Brodie et al., *Twenty First Century Science: GCSE Science Higher* (Oxford: OUP, 2006), p. 31.

26. M. Bailey et al., *AQA Biology for AS* (London: Hodder Education, 2011), p. 215.

27. WJEC AS Biology, Available at: http://www.wjec.co.uk/qualifications/science/as-a-level/human-biology-as-a-level-2009/GCE%20Biology-Human%20Spec%202009%20onwards%20Welsh%2028-04-14.pdf?language_id=1 (accessed 7 August 2017).

28. Edexcel GCSE Biology. Available at: https://qualifications.pearson.com/content/dam/pdf/GCSE/Science/2011/Specification%20and%20sample%20assessments/UG029988_GCSE_in_Biology_Spec_2012.pdf (accessed 7 August 2017).

29. AQA GCSE Biology. Available at: www.aqa.org.uk/subjects/science/gcse/science-a-4405/subject-content/unit-1-biology-2 (accessed 7 August 2017).

30. See www.dur.ac.uk/cells/engagement/scr/stream2/lec at 39:16–43.30. This was confirmed in email correspondence, before and following the lecture. For example, ‘I don’t know of any report in which nuclei were isolated from somatic cells’ (email correspondence with M. Herbert from 1 June 2015) (accessed 7 August 2017).

31. Wilmut et al., ‘Viable Offspring’, p. 810.

32. Wilmut et al., ‘Viable Offspring’, p. 813 (emphasis added).

33. H. Gee, ‘Dolly is Not Quite a Clone’, *Nature*, 31 August 1999. Available at: www.nature.com/news/1999/990831/full/news990902-5.html (emphasis added) (accessed 7 August 2017).

34. See, for example, D. Egli and G. Chia, ‘A Protocol for Embryonic Stem Cell Derivation by Somatic Cell Nuclear Transfer into Human Oocytes’, *Protocol Exchange* 2014. Available at: www.nature.com/protocolexchange/protocols/3117#/procedure: ‘Drill the zona and make a very small opening,…insert the somatic cell with the side exposed to the virus facing the plasma membrane, and ensure that the zona pellucida presses on the somatic cell to ensure contact between the somatic plasma membrane and the oocyte plasma membrane. If the somatic cell is GFP positive, fusion can usually be confirmed within 5 minutes by briefly checking the fluorescence in the oocyte’ (accessed 7 August 2017).

35. A. Trounson and N. D. DeWitt, ‘Pluripotent Stem Cells from Cloned Human Embryos: Success at Long Last’, *Cell Stem Cell* 12(6) (2013), p. 636, 638 (figure 1); D. P. Wolf et al., ‘Concise Review: Embryonic Stem Cells Derived by Somatic Cell Nuclear Transfer: A Horse in the Race?’, *Stem Cells* 35(1) (2017), p. 26, 28 (figure 1).

36. Trounson and DeWitt, ‘Pluripotent Stem Cells’, p. 638. Cf.; Tachibana et al., ‘Human Embryonic Stem Cells’, p. 1229: ‘mitochondrial DNA originated almost exclusively from oocytes’.

37. A YouTube video of which is available at: www.youtube.com/watch?v=oVaeFE0BMk8 (accessed 7 August 2017).

38. See D. Beyleveld and S. D. Pattinson, ‘Globalisation and Human Dignity: Some Effects and Implications for the Creation and Use of Embryos’, in R. Brownsword (ed.) *Global Governance and the Quest for Justice. Volume IV: Human Rights* (Oxford: Hart, 2004), p. 185; S. D. Pattinson, ‘Some Problems Challenging the UK’s Human Fertilisation and Embryology Authority’, *Medicine and Law* 24 (2005), p. 391.

39. CA, [44]–[49].

40. Pattinson, ‘Some Problems’, pp. 398–399.

41. M. Warnock et al., *The Report of the Committee of Inquiry into Human Fertilisation and Embryology* (London: HMSO, 1984), para 11.7.

42. On which, see A. Smajdor, *Background Paper: Artificial Gametes* (London: Nuffield Council on Bioethics, December 2015). Available at: http://nuffieldbioethics.org/wp-content/uploads/Background-paper-2016-Artificial-gametes.pdf (accessed 7 August 2017).

43. A. Grubb, ‘Regulating Cloned Embryos?’ *Law Quarterly Review* 118 (2002), p. 358; A. Plomer, ‘Beyond the HFE Act 1990: The Regulation of Stem Cell Research in the UK’, *Medical Law Review* 10 (2002), p. 132; D. Morgan and M. Ford, ‘Cell Phoney: Human Cloning After Quintavalle’, *Journal of Medical Ethics* 30 (2004), p. 524.

44. HL, [14]. Lords Hoffman and Scott agreed with Lord Bingham. See also the speeches of Lord Steyn (esp. [26]) and Lord Millet (esp. [45]–[47]).

45. Wilmut et al., ‘Viable Offspring’, p. 811.

46. On this second point, see also J. Montgomery and E. Montgomery, ‘Montgomery on Informed Consent: An Inexpert Decision?’ *Journal of Medical Ethics* 42 (2016), p. 89.

47. HL, [18].

48. Pattinson, ‘Some Problems’, pp. 397–398.

49. Available at: www.bera.ac.uk/researchers-resources/publications/ethical-guidelines-for-educational-research-2011 (accessed 7 August 2017).

50. Wilmut et al., ‘Viable Offspring’.

51. R. W. Ryan and H. R. Bernard, ‘Data Management and Analysis Methods’, in N. K. Denzin and Y. S. Lincoln, eds. *Handbook of Qualitative Research*. 2nd ed. (London: Sage Publications, 2000), pp. 769–802.

52. For example, AQA. Available at: www.aqa.org.uk/subjects/science/as-and-a-level/biology-2410 (accessed 7 August 2017).

53. An extended project qualification (EPQ) is a self-directed and self-motivated project that can be a research-based report, a production (e.g. musical show or concert) or an artefact (e.g. a computer game or piece of art). The EPQ is assessed via written report, presentation and production log. See www.aqa.org.uk/programmes/aqa-baccalaureate/extended-project/the-aqa-epq for further details (accessed 7 August 2017).

54. Other activities within our project had more success in this regard: see S. D. Pattinson, V. Kind and B. Douglas, *Ethics Activity Pack* (Durham: CELLS, 2017); S. D. Pattinson et al., *Stem Cell Research Activity Pack* (Durham: CELLS, 2017). Available at: www.dur.ac.uk/cells/packs (accessed 7 August 2017).

55. See, for example, E. Rackley, *Women, Judging and the Judiciary: From Difference to Diversity* (Abington, Oxon: Routledge, 2013).

